# Supplementary material for: Protein–protein interactions in the Mla lipid transport system probed by computational structure prediction and deep mutational scanning
Source: J Biol Chem. 2023 Apr 25;299(6):104744. doi: 10.1016/j.jbc.2023.104744 (PMC10245069; doi:10.1016/j.jbc.2023.104744)
Supplement: Supplementary Figures S1–S7 and Tables S1, S2, S4 [file mmc2.pdf]

Supporting Information

Protein-protein interactions in the Mla lipid transport system probed by computational structure prediction and deep mutational scanning

Mark R. MacRae*[,1], Dhenesh Puvanendran*[,1], Max A. B. Haase[1], Nicolas Coudray[1,2], Ljuvica Kolich[1], Cherry Lam[1], Minkyung Baek[3,4,#], Gira Bhabha[‡,1], Damian C. Ekiert[‡,1,5]

[1] Department of Cell Biology, New York University School of Medicine, New York, United States
[2] Applied Bioinformatics Laboratories, New York University School of Medicine, New York, United States
[3] Department of Biochemistry, University of Washington, Seattle, WA, USA.
[4] Institute for Protein Design, University of Washington, Seattle, WA, USA
[5] Department of Microbiology, New York University School of Medicine, New York, United States

*These authors contributed equally to this work
[‡]For correspondence: gira.bhabha@gmail.com (GB); damian.ekiert@ekiertlab.org (DCE)
[#]Present address: School of Biological Sciences, Seoul National University, Seoul, Republic of Korea

List of Contents Included in this File

Supplementary figure 1. Deep mutational scanning data statistics
Supplementary figure 2. Additional data for MlaC-MlaA predictions
Supplementary figure 3. Binding curve fits for biolayer interferometry experiments
Supplementary figure 4. Additional data for MlaC-MlaD predictions
Supplementary figure 5. Predicted protein-protein interactions of MlaC-MlaD in the outward state
Supplementary figure 6. Western blots for MlaD mutants
Supplementary figure 7. Cryo-EM data processing workflow and analysis
Supplementary Table 1: Cryo-EM data acquisition parameters
Supplementary Table 2: Cryo-EM processing information
Supplementary Table 4: Bacterial strains used in this study

Other Supporting Materials for this Manuscript

Supplementary Table 3: Plasmids used in this study
Supplementary Data File 1. Deep mutational scanning top hit statistics

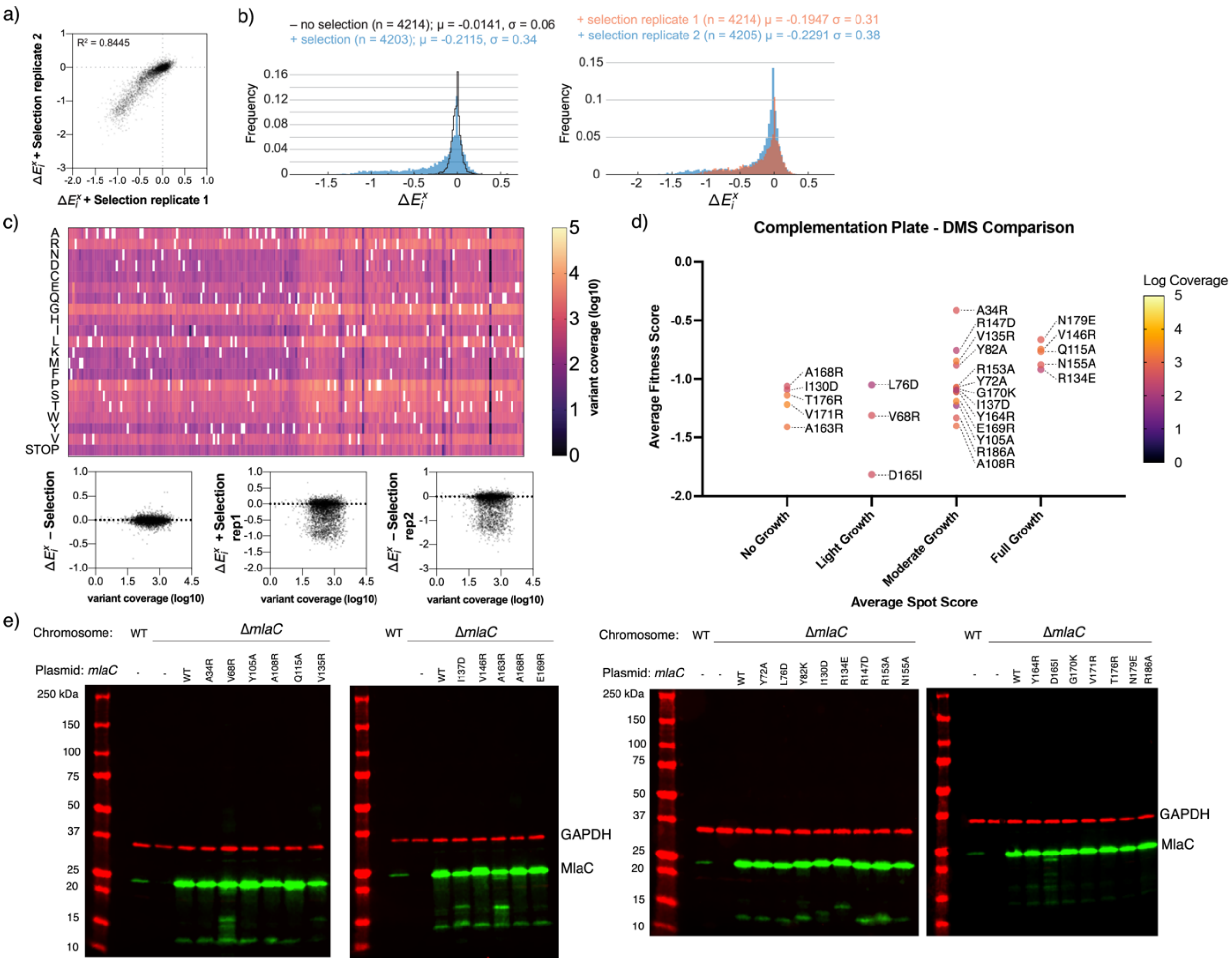

Supplementary figure 1. Deep mutational scanning data statistics. a) Relative fitness values of replicate 1 on x-axis and replicate 2 on y-axis give a R^2 of 0.8445, validating the reproducibility of the experiment. **b)** Two histograms detailing the spread in cellular fitness impact (on x-axis) across all mutations. y-axis represents frequency of occurrence; the relative number of mutations at a given cellular fitness impact can be observed. The left histogram details the average (between the two replicates) cellular fitness scores per mutation both with (blue) and without (black outline) selection. The average (μ) and standard deviation (σ) are shown for each. The right histogram shows the spread in cellular fitness impact for all mutations in the selection for replicate 1 (red) and replicate 2 (blue), again with the average (μ) and standard deviation (σ) shown for each. **c)** Heat map of coverage for each mutation made on MlaC. Overall, each mutant is at least represented 100 times. White rectangles represent the residues corresponding to the WT sequence of MlaC. Coverage vs fitness plots, indicate that apparent fitness is largely unaffected by variant coverage. **d)** Plot correlating the average (n=3) MlaC point mutant growth by complementation assay against the average fitness score of the mutation by deep mutational scanning (n=2). Growth in complementation assays were scored in one of four categories: “No Growth” indicating similar growth to MlaC KO; “Light Growth” indicating partial growth more similar to *mlaC* KO than to WT; “Moderate Growth” indicating partial growth more similar to WT than to *mlaC* KO; and “Full Growth” indicating bacteria growth similar to the WT. Each mutation is colored according to the Log read coverage. **e)** Western blots of MlaC mutants used in genetic complementation experiments. Green bands correspond to MlaC (measured at 800 nm) and red bands correspond to the GAPDH loading control (measured at 680 nm).

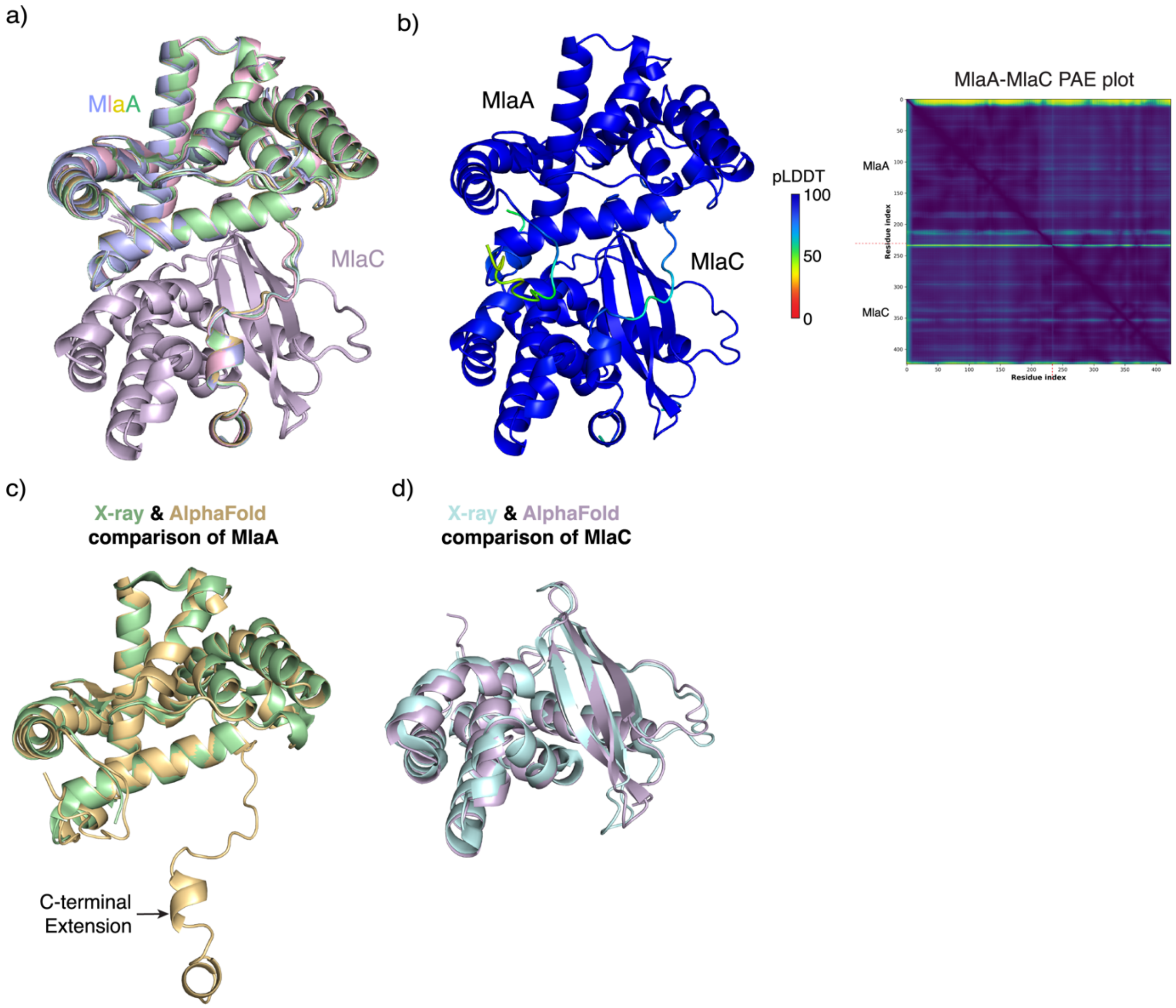

Supplementary figure 2. Additional data for MlaC-MlaA predictions. a) The 5 different 1:1 MlaC:MlaA AlphaFold Multimer predictions show a high similarity between each model (model 1, gold; model 2, green; model 3, blue; model 4, pink; model 5, cyan). Models have been aligned on MlaC, shown in purple. **b)** Confidence statistics for the MlaA-MlaC AlphaFold Multimer prediction are shown with pLDDT values colored as in the key on the predicted complex and the predicted alignment error (PAE) values between the two proteins plotted on a heatmap. **c)** Superimposition of MlaA from predicted model 1 (gold) and the crystal structure of MlaA (green; PDB 5NUO). **d)** Superimposition of MlaC from predicted model 1 (purple) and the crystal structure of MlaC (cyan; PDB code 5UWA).

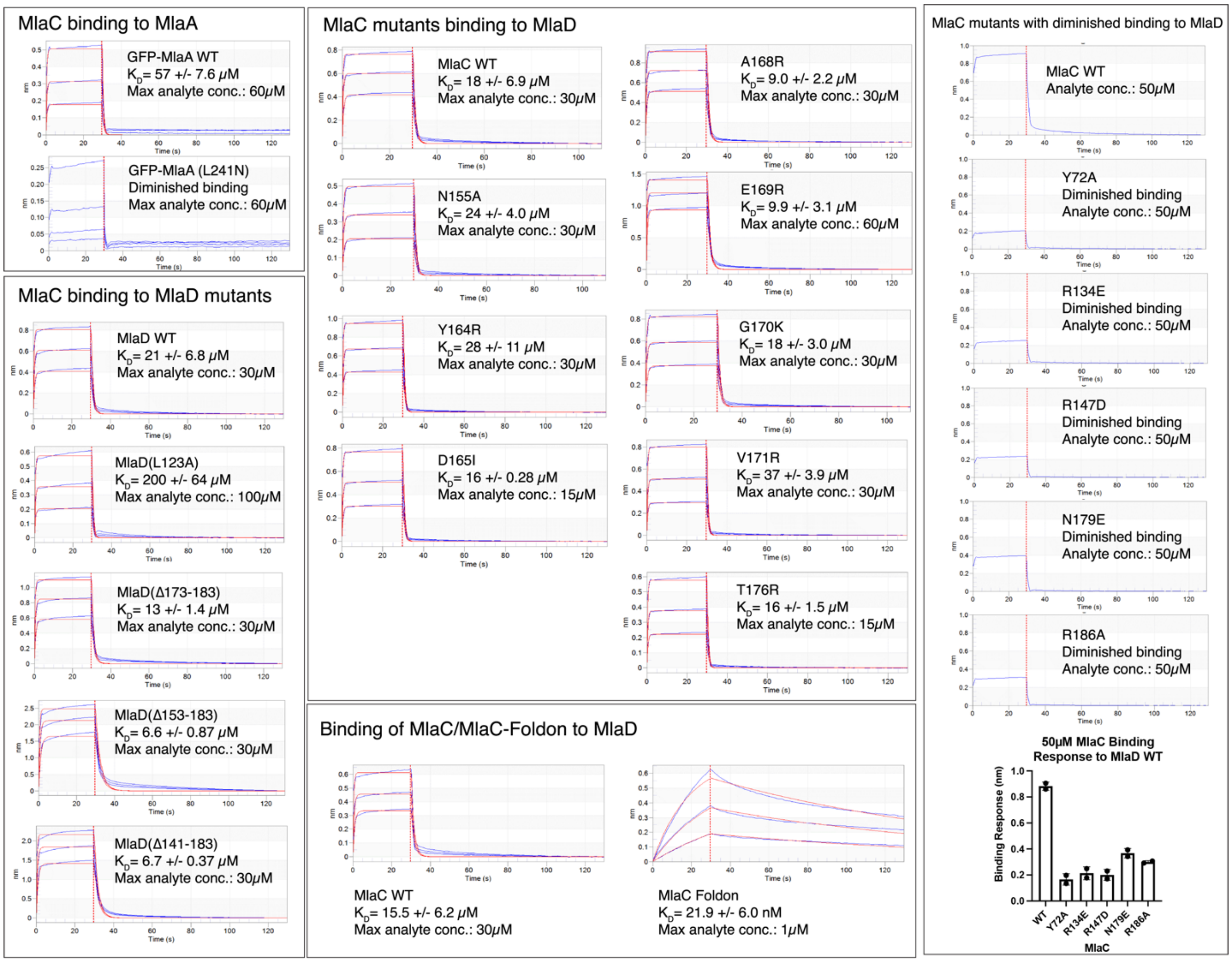

Supplementary figure 3. Binding curve fits for biolayer interferometry experiments. Curves for full titration (blue) and fits (red), for each binding interaction. A 1:1 binding model was used to generate a binding constant (K_D, μM) with an error between experiments (n=2). Binding curves for MlaC mutants (50 μM) that have diminished binding against MlaD WT are all shown at the same scale and relative to the curve for MlaC WT (50 μM). Additionally, a plot with the binding response of 50 μM MlaC proteins against MlaD WT is shown.

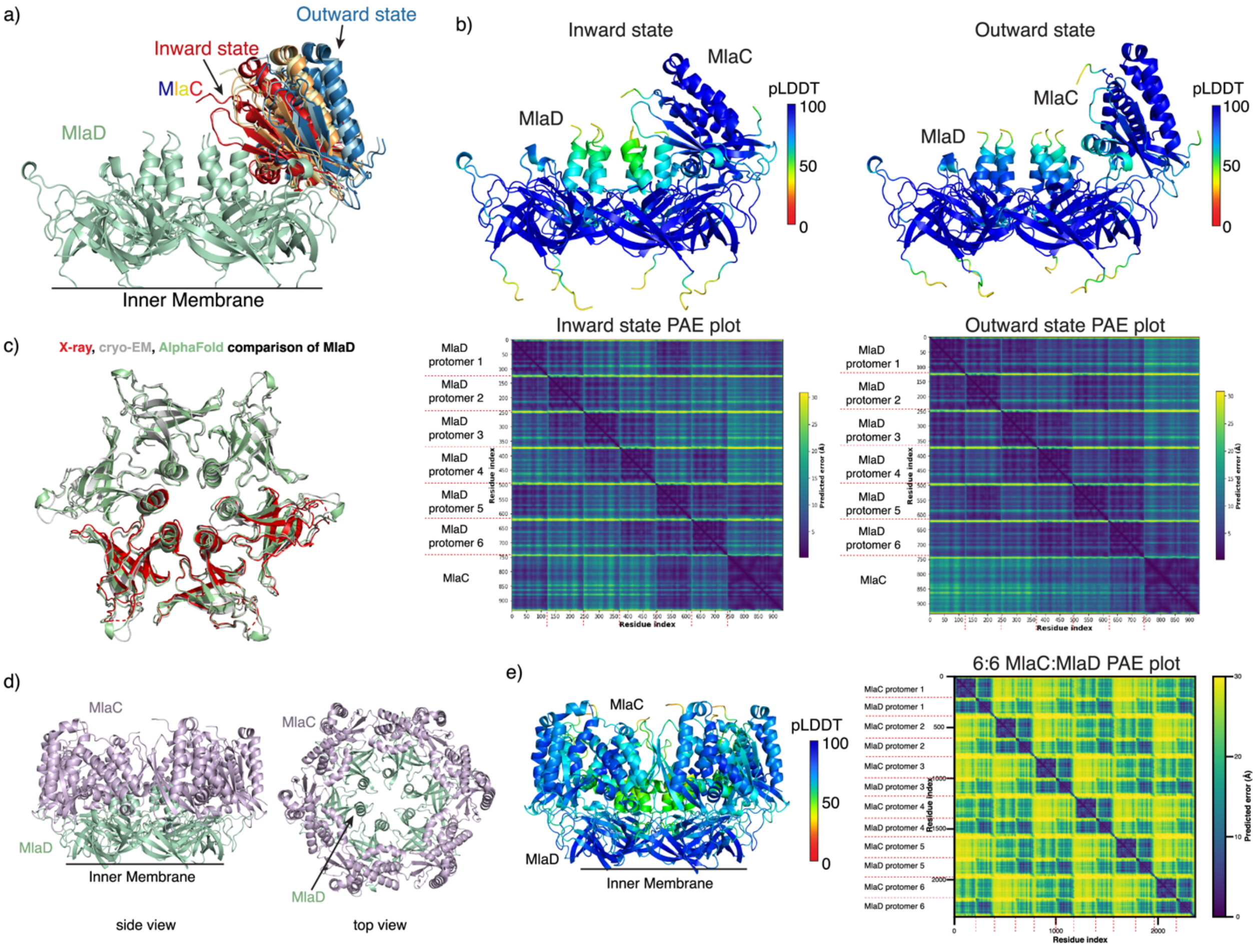

Supplementary figure 4. Additional data for MlaC-MlaD predictions. a) The 5 different 1:6 MlaC:MlaD predictions are shown with the 5 MlaC molecules colored from red to orange, yellow, light blue, and blue as they go from inward-facing to most outward facing states. Models are aligned on the MlaD ring. The inner membrane is indicated as a black line below MlaD. **b)** Confidence statistics for the inward and outward state AlphaFold2 predictions are shown. The pLDDT values are shown colored as in the key on the predicted models. The PAE values between chains in both predictions are plotted on a heatmap. **c)** Superposition of predicted MlaD from model 1 (green) with the crystal structure (red; PDB 5UW2), and the cryo-EM structure (gray; PDB 6XBD). **d)** The 6:6 MlaC:MlaD prediction; side and top facing views, with each MlaC colored in purple MlaD in green. One MlaC is observed to interact with the outer loop of one protomer and remain oriented roughly perpendicular to MlaD. The top five predicted models are consistent with each other. The inner membrane is indicated as a black line below MlaD. **e)** Confidence statistics for the MlaC-MlaD 6:6 AlphaFold2 prediction are shown with pLDDT values colored as in the key on the predicted complex and the predicted alignment error (PAE) values between the proteins plotted on a heatmap.

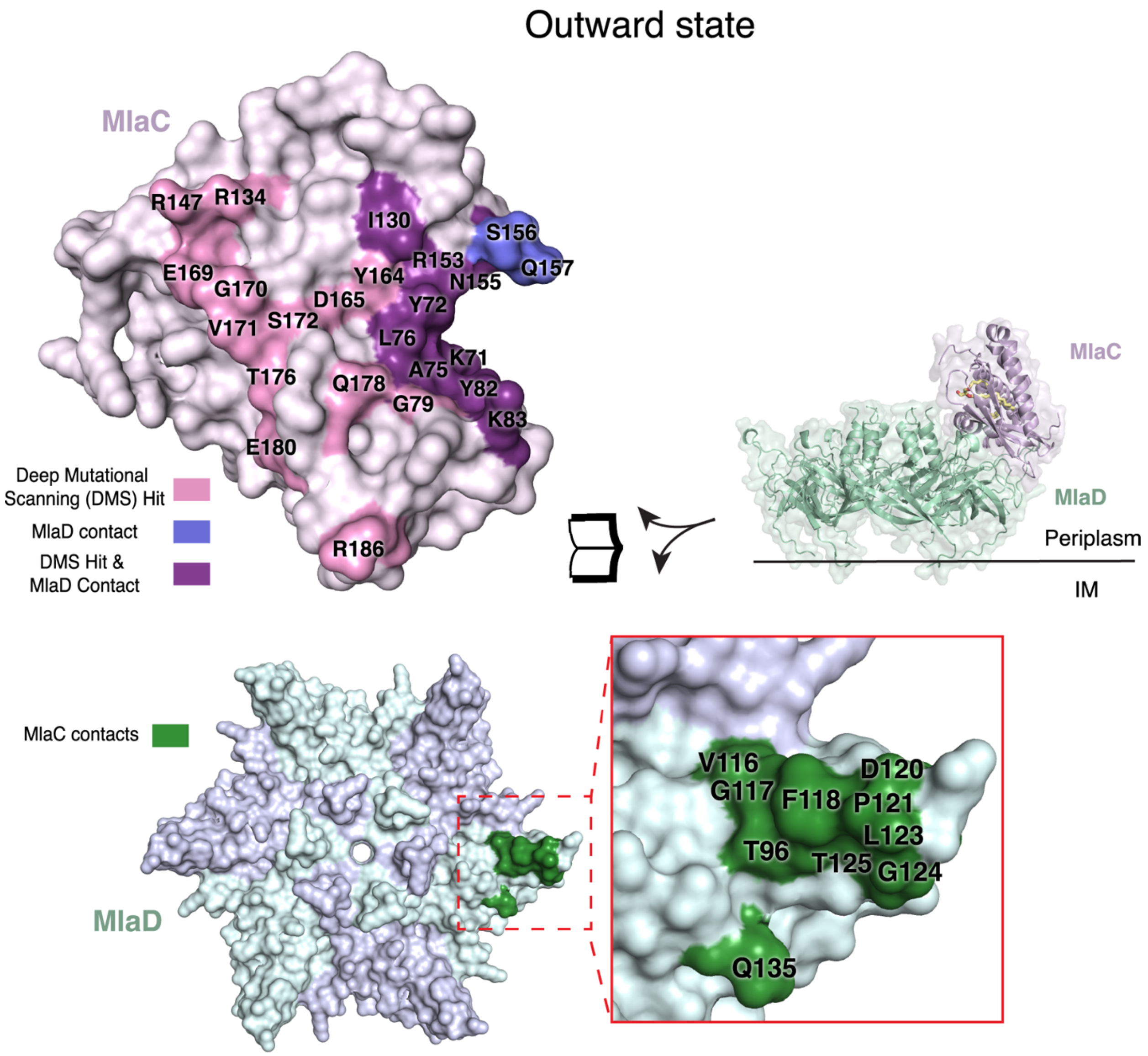

Supplementary figure 5. Predicted protein-protein interactions of MlaC-MlaD in the outward state. MlaC and MlaD are rotated as indicated and shown as molecular surfaces, highlighting interaction interfaces in the inward state. Residues in MlaC predicted to be within 4 Å distance of MlaD from AlphaFold2, shown to have reduced fitness from deep mutational scanning (DMS), or both, are mapped onto the structure as indicated in the key. Residues in MlaD predicted to be within 4 Å distance of MlaC from AlphaFold2 are mapped onto the structure as indicated in the key.

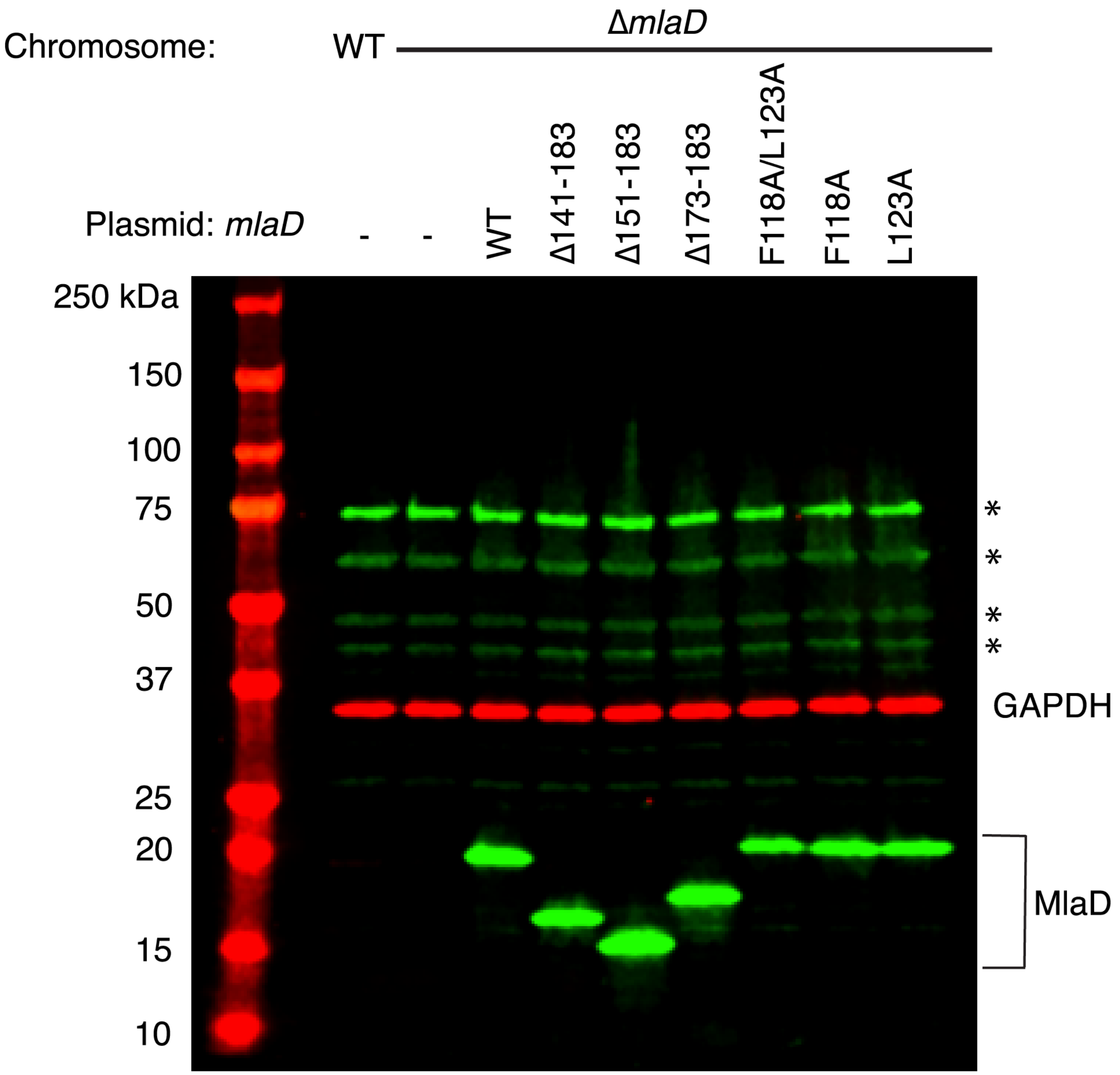

Supplementary figure 6. Western blots for MlaD mutants. a) Western blot again MlaD, corresponding to mutants used in genetic complementation experiments. Green bands correspond to MlaD (measured at 800 nm) and red bands correspond to the GAPDH as a loading control (measured at 680 nm). Asterisks correspond to non-specific bands present in all strains, including the Δ*mlaD* strain. Signal for MlaD in the WT strain is only marginally above background, and is highly over-expressed in complemented strains.

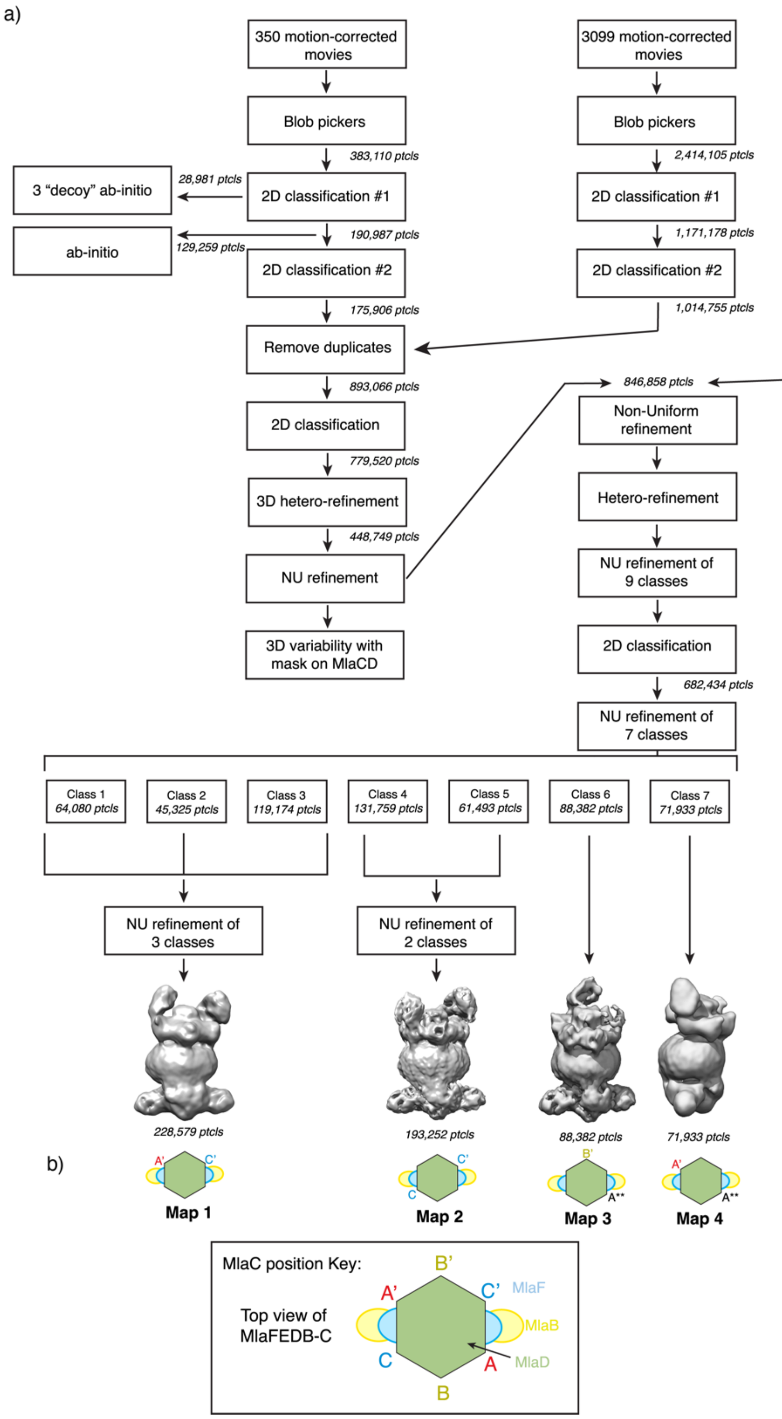

c)

AlphaFold Model-CryoEM Map CC Values

		MlaD		MlaC	
		Inward	Outward	Inward	Outward
**Map 1**	Site C'	0.5935	0.6368	0.3366	0.6502
	Site A'	0.6050	0.6397	0.2700	0.5773
**Map 2**	Site C'	0.6276	0.6568	0.2703	0.7229
	Site C	0.6293	0.6508	0.2219	0.5217
**Map 3**	Site B'	0.6977	0.7225	0.2406	0.7509
	Site A**	0.6871	0.7143	0.2034	0.5723
**Map 4**	Site A'	0.7590	0.7976	0.3172	0.7457
	Site A**	0.7444	0.7825	0.1220	0.5664

Supplementary figure 7. Cryo-EM data processing workflow and analysis. a) Cryo-EM data processing workflow. **b)** Schematic showing which chain of MlaD is primarily interacting with MlaC. MlaFEDB is represented with MlaD as a green hexagon, MlaF and MlaB in blue and yellow, respectively. Based on a previous high-resolution structure (PDB 6XBD) (14), Chains A and A' correspond to the MlaD monomers in which the MlaD transmembrane helices are tightly interacting with MlaE, chains B and B' are those observed to interact with the C-terminus of the interfacial helix IF1 helix of MlaE, and chains C and C' interact closely with the N-terminus of this helix. **c)** CC values of the MlaC-D AlphaFold2 predictions of the inward and outward states fit into the four final maps.

Supplementary Table 1: Cryo-EM data acquisition parameters.

Microscope	Arctica
Voltage (kV)	200
Nominal magnification	36,000
Detector	K3
Pixel size (Å/pix)	0.548 (super-resolution)
No. of micrographs	3,449 untilted and 3,875 tilted (40 deg)
No. of frames	40 and 48
Total dose (e-/Å^2)	56.70 and 50.78
Exposure time (s)	2.8 sec and 2.4 sec
Defocus range (μm)	1.5-2.9 and 1.1-2.6

Supplementary Table 2: Cryo-EM processing information.

Software	CryoSparc V3			
Total no. of particles in the final 4 classes	582,146			
Box size (pixels)	304			
Map	Map 1	Map 2	Map 3	Map 4
Symmetry	C1	C1	C1	C1
Number of particles	228,579	193,252	88,382	71,933
Map sharpening B factor (Å^2)	None	None	None	None
Resolution range based on CryoSPARC local resolution map (Å)	5-12	5-10	5-10	5-12
EMDB	EMD-40162			
Coordinates	rigid-body docking of AlphaFold2 models (Chimera)			

Supplementary Table 4: Bacterial strains used in this study

Strain ID	Genotype	Source
BW25113	"WT"; Δ(*araD-araB*)567 Δ(*rhaD-rhaB*)568 Δ*lacZ4787*(::*rrnB-3*) *hsdR514 rph-1*	Coli Genetic Stock Center
JW3160	BW25113 Δ*mlaD* (Keio background)	Baba 2006
JW2343	BW25113 *ΔmlaA* (Keio background)	Baba 2006
JW3159	BW25113 *ΔmlaC* (Keio background)	Baba 2006
bBEL182	BW25113 Δ*mlaD*	Coudray 2020
bBEL465	BW25113 *ΔmlaA*	This study
bBEL464	BW25113 *ΔmlaC*	This study

Supplementary Data File 1. Deep mutational scanning top hit statistics. The “top hits” for positions in MlaC that are most sensitive to mutation. A position in MlaC was considered a hit if 5 or more amino acid substitutions at that position resulted in a fitness decrease of more than one standard deviation below the average fitness of all mutations. Residues are grouped by their location on MlaC, including: SS (signal sequence), buried within the structure, in the lipid binding pocket, or exposed to the solvent. Included are the average fitness costs (between 2 replicates of deep mutational scanning) across all mutations for a residue and the standard deviation of this average. Additionally, point mutants assessed from this group to qualify the deep mutational scanning data are shown along with the average fitness cost for that specific mutation between the two replicates and the complementation spot plate result. Finally, MlaC residues that are predicted to interact with MlaD or MlaA based on our AlphaFold2 predictions (within 4 Å contact distance) are indicated.
